# Treatment of Typhoid Fever

**Published:** 1889-07

**Authors:** John D. Skeer

**Affiliations:** Chicago


### TREATMENT OF TYPHOID FEVER*

BY JOHN D. SKEER, M. D., CHICAGO.

Before we commence the treatment of a case of typhoid fever we should make ourselves acquainted with the family history, the clinical history, and the immediate sanitary surroundings of the patient. We will then be prepared to anticipate any compli-

*Read at the April meeting of the Chicago Pathological Society.

cations that might arise during the progress of the disease, and to treat the case understandingly.

A typical uncomplicated case of typhoid fever, as we find it in the mountainous regions of Virginia, Maryland, Pennsylvania, and the New England States, would seem to be a very different disease from that we encounter on our Western prairies, where it partakes more of the typho-malarial type, the malarial element predominating during the early stage of the disease, and the period of invasion only three or four days; whereas in the *true* type the period of invasion, before the patient takes his bed, is from two to three weeks duration.

Trousseau writes that "to know the course of disease, is more than half of medicine,"—an observation which applies with peculiar emphasis in typhoid fever. To know the course of typhoid fever is more than half the cure.

If I were to formulate a rule for the treatment of typhoid fever, I should write: *Quiet, sleep, nutriment.*

Watson says that "a case of typhoid fever that has been without sleep for four days and nights will surely die." Nutriment to sustain the vital forces is imperatively demanded, and should be given at stated intervals whether the patient desires it or not. *Milk* is the only article of diet that will support the system during these protracted fevers.

A young, robust Scotchman, who was brought home from Texas in a state of delirium, lived on buttermilk exclusively for three weeks. His delirium was so violent that it was suggested by his friends that he be taken to an insane asylum. Hydrate of chloral and bromide of potash quieted him so that he was able to recognize his friends the next day. Calomel is the smoothest physic that can be given in any form of typhoid fever. You cannot salivate a typhoid fever patient until after the fever has subsided—then you don't need to.

*Quinine* is indispensable in the malarial form, in the early stage of the disease,—pushed to a slight cinchonism, when it should be discontinued, to be resumed, if necessary, to arrest periodical chills. It is well to anticipate the recurrence of chills with quinine once a week.

In the true typhoid type, when the tongue is dry, fissured, cracked and bleeding, quinine is not only contraindicated, but it is positively injurious. In such cases I give carbolic acid and iodine after the formula of Bartholow, and sulphurous acid between each dose.

I do not use antipyrin in typhoid fever, for the reason that it retards the processes of oxidation. The half oxidized material is not eliminated, but is retained in the blood and poisons the brain and nerve centers. It may reduce the temperature, but it is far from being a curative agent in typhoid fever. On the contrary, I believe it to be injurious.

I was called to see a case where the antipyrin had been given in doses of from 30 to 40 grains daily for two weeks. The temperature was 106°. The patient died promptly.

*Water* is the antipyretic—given internally and applied to the surface of the body when the skin is hot and dry. The wet pack is the best method of applying the water; but the people are afraid of it. I always pack the abdomen with towels wrung out of cold water to be bound on smoothly with a flannel bandage, and changed every hour or two.

A Dublin physician years ago reported a large number of cases treated successfully with sulphurous acid, which is one of our best germicides. He did not know that he had a typhoid bacillus and pus-microbe to annihilate or paralyze before recovery could take place.

To arrest the diarrhœa I usually prescribe chalk mixture, subnitrate of bismuth, saccharated pepsin, and McMunn's elixir in combination. McMunn's elixir of opium is the only preparation that can be given with safety in typhoid fever. Morphine, if given to produce sleep, should be given in sedative doses. Small doses stimulate the brain and make the patient wakeful. I prefer chloral and bromide of potash. They do not dry up the secretions, disturb the brain, or offend the stomach like the preparation of opium.

In the advanced stages of the disease, if the temperature should fall one or two degrees below normal, send immediately for medicine to arrest hemorrhage of the bowels, for you will be most certain to require it before the messenger has had time to return. This sudden fall in temperature may escape our observation until after the hemorrhage has taken place. The temperature will rise again, but not so high as before, unless it is a few hours before dissolution, when it usually runs up to 106–7°.

My last case of hemorrhage of the bowels yielded very readily to teaspoonful doses of the extract of hamamelis, given every fifteen or twenty minutes, together with hot clove tea and brandy. The hot pack was applied over the abdomen. The patient made a good recovery. The hamamelis seems to pass into the bowels more readily than any of the other astringents.

We should never for one moment lose sight of the fact that the greatest danger in typhoid fever arises from perforation of the bowels. The typhoid bacillus finds its nidus in the glands of Peyer and Brünner, and excites (with the assistance of the pus microbe) irritation, inflammation and ulceration, which may perforate the coats of the bowels and give rise to fatal peritonitis.

The patient should sleep and have abundant nutriment to support the brain and nervous system. The bowels should be kept quiet to give time for the ulcers to heal.

